# LncRNA lnc‐ISG20 promotes renal fibrosis in diabetic nephropathy by inducing AKT phosphorylation through miR‐486‐5p/NFAT5

**DOI:** 10.1111/jcmm.16280

**Published:** 2021-05-03

**Authors:** Yu‐Rui Duan, Bao‐Ping Chen, Fang Chen, Su‐Xia Yang, Chao‐Yang Zhu, Ya‐Li Ma, Yang Li, Jun Shi

**Affiliations:** ^1^ Department of Nephrology Huaihe Hospital of Henan University Kaifeng China; ^2^ Department of Urology Huaihe Hospital of Henan University Kaifeng China

**Keywords:** AKT, diabetic nephropathy, fibrosis, Lnc‐ISG20, MicroRNA‐486‐5p, NFAT5

## Abstract

Long non‐coding RNA (lncRNA) lnc‐ISG20 has been found aberrantly up‐regulated in the glomerular in the patients with diabetic nephropathy (DN). We aimed to elucidate the function and regulatory mechanism of lncRNA lnc‐ISG20 on DN‐induced renal fibrosis. Expression patterns of lnc‐ISG20 in kidney tissues of DN patients were determined by RT‐qPCR. Mouse models of DN were constructed, while MCs were cultured under normal glucose (NG)/high glucose (HG) conditions. The expression patterns of fibrosis marker proteins collagen IV, fibronectin and TGF‐β1 were measured with Western blot assay. In addition, the relationship among lnc‐ISG20, miR‐486‐5p, NFAT5 and AKT were analysed using dual‐luciferase reporter assay and RNA immunoprecipitation. The effect of lnc‐ISG20 and miR‐486/NFAT5/p‐AKT axis on DN‐associated renal fibrosis was also verified by means of rescue experiments. The expression levels of lnc‐ISG20 were increased in DN patients, DN mouse kidney tissues and HG‐treated MCs. Lnc‐ISG20 silencing alleviated HG‐induced fibrosis in MCs and delayed renal fibrosis in DN mice. Mechanistically, miR‐486‐5p was found to be a downstream miRNA of lnc‐ISG20, while miR‐486‐5p inhibited the expression of NFAT5 by binding to its 3'UTR. NFAT5 overexpression aggravated HG‐induced fibrosis by stimulating AKT phosphorylation. However, NFAT5 silencing reversed the promotion of in vitro and in vivo fibrosis caused by lnc‐ISG20 overexpression. Our collective findings indicate that lnc‐ISG20 promotes the renal fibrosis process in DN by activating AKT through the miR‐486‐5p/NFAT5 axis. High‐expression levels of lnc‐ISG20 may be a useful indicator for DN.

## 1 INTRODUCTION

Diabetic nephropathy (DN), also known as diabetic kidney disease, affects the normal functioning of kidneys in approximately 50% of all the patients suffering from both type I and type II diabetes.^1^, ^2^, ^3^ Furthermore, these diabetic patients are highly susceptible to macrovascular complications.^1^ The hard‐done work of our peers has revealed that DN is precipitated by multiple factors, such as glucose metabolism disorder, genetic susceptibility, renal hemodynamic changes and so on; however, the actual mechanisms underlying DN remain to be elucidated.^4^ Pathologically, DN is characterized by changes in renal function and structure, while such aberrations can lead to renal dysfunction, renal fibrosis, podocyte injury, as well as the end‐stage renal disease (ESRD).^5^ Nowadays, microalbuminuria is considered as the ‘gold‐standard’ for the diagnosis of DN, and the patients presenting with appearance of microalbuminuria would further progress into significant proteinuria, impaired renal function and even ESRD, which is particularly alarming.^6^ Although microalbuminuria can serve as a clinical marker for DN, it lacks sufficient sensitivity and specificity for the early manifestations of the disease.^7^ On the other hand, the degree of renal fibrosis is also considered to be a key indicator of worsening renal function as well as the core of high mortality of DN.^8^ Renal fibrogenesis is primarily caused by the accumulation of extracellular matrix (ECM) proteins such as collagen and fibronectin, as well as epithelial‐to‐mesenchymal transition.^9^ Activation of mesangial cells (MCs), major cellular constituents in glomerular mesangium, participates in process of renal glomerular fibrosis through inducing hyperproliferation and excess ECM.^10^ Currently, the available therapies are not fully efficacious in the treatment of DN, therefore, identification of the underlying mechanisms as well as diagnostic and prognostic hallmarks for DN or renal fibrosis could be of great value for better management of DN.

In recent years, a large number of studies have suggested that microRNAs (miRNAs), a kind of short non‐coding RNA molecules with ~19‐22 nt in length, are implicated in the pathogenesis of DN or related complications, indicating that miRNAs possess great potential in the diagnosis of early state diseases.^11^, ^12^, ^13^ Moreover, numerous miRNAs, such as miR‐21, miR‐34a‐5p, miR‐141, miR‐25 and miR‐486‐5p, have been previously documented to be dysregulated in DN,^7^, ^11^ among which the differential expression of miR‐486‐5p in the patients with DN was also verified to be significantly correlated with albuminuria and blood glucose or lipid abnormalities.^7^, ^14^ Elucidation of the signalling pathways mediated by miRNAs could accelerate the development of valuable biomarkers for DN.

In addition to miRNAs, long non‐coding RNAs (lncRNAs), which belong to a class of non‐coding RNAs with >200 nt in length, have also been reported to be involved in multifarious physiological and pathological processes, such as cell cycle progression, cell proliferation, chromatin modulation.^15^, ^16^, ^17^ In addition, lncRNAs can bind to various molecules such as DNA, RNA or proteins to regulate different processes, including transcription, mRNA stabilization and protein translation.^15^ So far, very little is known about the role of lncRNAs or miRNAs in regard to renal fibrosis of DN, especially the related signalling pathways. Therefore, investigation of the underlying mechanisms for renal fibrogenesis, particularly the miRNA‐mediated signalling, may provide valuable insights into the diagnosis and treatment of DN.

Additionally, the nuclear factor of activated T cells (NFAT) represents a kind of Ca^2 +^ ‐dependent transcription factors, which is the substrate for calcineurin (CaN).^18^ Despite that NFAT was initially considered to be restricted within the immune system, a growing number of researches have shown that NFAT can be expressed in non‐immune cells to affect various physiological processes, including renal tubular cell apoptosis and glomerulosclerosis, whereas inhibition of NFAT‐related pathway can ameliorate DN at the early stage.^19^, ^20^ Moreover, several studies have revealed the involvement of AKT in DN, and AKT is generally activated by cytokines.^21^ More importantly, regulation of AKT‐mediated inflammation has been previously suggested to modulate the renal function during DN.^22^ Moreover, AKT activation during hyperglycemia directly correlates with renal dysfunction.^23^ Therefore, further investigation of the roles of NFAT and AKT in DN pathogenesis may help deepen our understanding of DN.

Recently, the lncRNA lnc‐ISG20 was found to be up‐regulated in the glomerular in DN patients,^24^ but the actual mechanism remains unclear, based on which we set out to elucidate the molecular mechanism of lnc‐ISG20 in regulating the progression of renal fibrosis in DN. In this study, our data revealed that knockdown of lnc‐ISG20 could inhibit the renal fibrosis of DN, and lnc‐ISG20 could inhibit the expression of miR‐486‐5p which was further found to be able to inhibit the AKT phosphorylation *via* NFAT5. Here, these results provide evidence for that lnc‐ISG20 could promote the renal fibrosis in DN *via* miR‐486‐5p/NFAT5/AKT.

## 2 MATERIALS AND METHODS

### 2.1 Ethics statement

Signed informed consents were obtained from all participants or their families prior to the study, and each experimental procedure was approved by the Huaihe Hospital of Henan University Ethics Committee. All animal experiment procedures were strictly in accordance with the recommendations in the laboratory animal care and use guidelines published by the National Institutes of Health. Extensive efforts were made to minimize the suffering of the animals included in the study.

### 2.2 Tissue collection

The kidney tissues were collected from 30 patients diagnosed as DN by renal biopsy at the Huaihe Hospital of Henan University from January 2018 to March 2019. The patients comprised of 15 males and 15 females, with a calculated mean age of 54.93 ± 10.17 years. In addition, adjacent normal kidney tissues taken from patients with kidney cancer served as controls, which included 15 males and 15 females, with a calculated mean age of 52.20 ± 8.24 years. The patients with renal cancer in the control group presented with normal renal function, blood glucose and urine protein before surgery, and no history of heart or liver disease.^25^

### 2.3 Mouse model of DN

C57BL/KsJ db/db male mice (aged 8‐weeks) and their age‐matched db/m mice were purchased from NBRI (Nanjing, China). Leptin receptor (db/db) gene‐deficient mice are widely used to establish type 2 diabetes models. Similarly, db/db mouse can be employed to construct a model of human DN, as they exhibit main common characteristics similar to humans such renal hypertrophy, glomerular enlargement, proteinuria and mesangial matrix expansion. Several studies have also confirmed that db/db mice exhibit hyperglycemia at 4 weeks of age, and the albumin excretion rate is 8‐62 times higher at 8 weeks of age.^26^ Therefore, 8‐week‐old db/db mice were regarded as the early stage of DN mice and used for experimentation in the current study. The mice in the DN group were continuously fed with high‐glucose (HG) and high‐fat diet, while the mice in the control group were fed with normal diet. All mice were kept in a 12‐hours light/dark cycle in a non‐pathogenic environment with free access to food and water. Three weeks after treatment, all mice were euthanized after anaesthesia by intraperitoneal injection of 2% pentobarbital sodium,^27^ and then both kidneys were quickly and accurately removed from mice to study. A portion of the tissues was fixed in 4% paraformaldehyde, paraffin‐embedded and stored at −80°C, and the remaining tissues were stored in liquid nitrogen and used for Western blot assay.^28^

### 2.4 Animal treatment

The mice were assigned into control group, DN group, short hairpin RNA (sh)‐negative control (NC) group, sh‐lnc‐ISG20 group, overexpression (oe)‐NC group, oe‐lnc‐ISG20 group, oe‐NC + sh‐NC group, oe‐lnc‐ISG20 + sh‐NC group, oe‐NC + sh‐NFAT5 group, oe‐lnc‐ISG20 + sh‐NFAT5 group, 10 mice per group. Two weeks before HG treatment, mice were injected with adeno‐associated virus (AAV)2/2 vector expressing oe‐lnc‐ISG20, sh‐lnc‐ISG20, sh‐NFAT5 and AAV‐NC alone or in combination through tail vein.

### 2.5 Biochemical analysis

A Bayer blood glucose metre was used to measure blood glucose, and the Coomassie brilliant blue method was applied to detect the 24‐hour urine albumin concentrations.^28^

### 2.6 Haematoxylin‐Eosin (HE) staining

The collected kidney tissues were dehydrated with fixed gradient alcohol (70%, 80%, 90%, 100%), paraffin‐embedded, and then sliced into 4 μm thick sections. The sections were then placed in an oven at 60°C with 1 hour, dewaxed with conventional xylene and dehydrated with gradient alcohol. The sections were stained with haematoxylin (H8070, Solarbio Technology Co., Ltd., Beijing) for 3 minutes and eosin (Shanghai Sangon Biotech Co., Ltd., Shanghai, China) for 1 minute. The sections were returned to blue with flow water for 5 minutes, dried, sealed with neutral gum, observed and imaged under an optical microscope (XSP‐36, Boshida Optical Instrument Co., Ltd., Shenzhen, China). A total of 5 high‐magnification fields were randomly selected for each slice (× 200).^29^

### 2.7 Masson staining

The collected kidney tissues were dehydrated with fixed concentrations of gradient alcohol (70%, 80%, 90%, 100%), paraffin‐embedded, and then sliced into 4 μm thick sections. The obtained sections were then placed in an oven at 60°C with 1 hour, dewaxed with conventional xylene and dehydrated with gradient alcohol. After hydration, steps were performed according to the Masson staining kit (G1340, Beijing Solarbio Technology Co., Ltd., Beijing, China). After Reguad staining for 5 minutes, and colour separation in picric acid ethanol solution, the sections were rinsed once with double distilled water. After dyeing with Ponceau red acid for 5 minutes, the sections were rinsed in double distilled water once, and immersed in l% phosphoaluminate for 5 minutes, aniline acetate blue solution for 7 minutes, and 1% acetic acid for 1 minute, in successive. After 95% ethanol dehydration, anhydrous ethanol dehydration and xylene clearing, the sections were sealed with neutral gum. Results of Masson staining were used to determine the collagen deposition in rat renal tubules and the proportion of total kidney collagen area. Digital images of kidney tissue were acquired using an optical microscope (Olympus) equipped with an image analysis software (Image‐Pro Plus version 6.0; Media Cybernetics, Bethesda, MD, USA). Five fields were selected for each slice, and the area of fibrotic lesions in the cortical interstitium was calculated in any field and showed as a percentage of the area of fibrosis relative to the entire area.^30^

### 2.8 Immunohistochemistry

The methods of fixing, dehydrating, slicing, baking, dewaxing and hydrating were as stated above. The sections were repaired with 0.1 M sodium citrate, heated and boiled for 20 min. After being allowed to cool down, the sections were washed with 0.2 mol/L PBS solution (pH 7.4) for 5 minutes × 3 times. Next, 3% catalase was added for 15 minutes reaction, and 0.2 mol/L PBS solution was used to rinse for 5 minutes × 3 times. With the addition of 5% BSA blocking solution dropwise, the sections were incubated at 37°C for 30 minutes. Primary antibodies from Abcam (Cambridge, UK), rabbit anti‐collagen IV (1:500, ab6586, Abcam), rabbit anti‐fibronectin (1:500, ab2413, Abcam) and rabbit anti‐TGF‐β1 (1:500, ab92486, Abcam) were added dropwise for incubation overnight at 4°C in a wet box. The sections were washed with 0.2 mol/L PBS solution for 5 minutes × 3 times. After drying the sections, the biotinylated goat anti‐rabbit IgG (1:1000, ab6721, Abcam, Cambridge, UK) was incubated with the sections at 37°C for 30 minutes. Following incubation with horseradish peroxidase‐labelled streptavidin protein working solution (DA1010, Solable Technology Co., Ltd., Beijing) at 37°C for 20 minutes, the sections were washed with 0.2 mol/L PBS for 5 minutes × 3 times. The sections were counterstained with haematoxylin, after which 1% ammonia was used to return to blue. After dehydration, transparency and sealing, tissue sections were observed under an optical microscope (XSP‐36, Boshida Optical Instrument Co., Ltd., Shenzhen, China). A total of 5 high‐magnification fields were randomly selected for each section. 100 cells were counted in each field, where the number of positive cells <5% was considered as negative, and the number of positive cells ≥5% was positive. The results of immunohistochemistry were independently scored by two pathologists in a double‐blind manner.^29^

### 2.9 Cell culture

Human embryonic kidney cells HEK293T and mouse glomerular mesangial cells (MCs) were purchased from the Shanghai Academy of Sciences (Shanghai, China). The obtained HEK293T cells were cultured with HG DMEM (10 564 029, Gibco, USA), and MCs were cultured with low‐glucose DMEM (10 567 022, Gibco, USA). Next, 10% foetal bovine serum (16 000 044, Gibco, USA), 10 μg/mL streptomycin and 100 U/mL penicillin were cultured in an incubator (Thermo Co., USA) under the conditions of 37°C, 5% CO_2_ concentration and 95% saturation humidity. The MCs in the normal glucose (NG) group were cultured with L‐glucose 30 mmol/L, and those in the HG group were cultured with D‐glucose 30 mmol/L. HG stimulation simulated the growth environment of MCs under DN conditions, and NG stimulation simulated the normal growth environment.^28^

### 2.10 Cell transfection

Cells were digested with trypsin in the logarithmic phase and inoculated with 6‐well plates at 1 × 10^5^ per well. After 24 hours, Lipofectamine^TM^ 2000 (Invitrogen Carlsbad, CA) was used to transfect sh‐NC (5′‐GGGUGAACUCACGUCAGAA‐3′ sequence), sh‐lnc‐ISG20‐1 (5′‐CCAGGUGUCGAUAAGUAAUTT‐3′ sequence), sh‐lnc‐ISG20‐2 (5′‐CUGUAAGUAGAUCCUGCAGTT‐3′ sequence), sh‐NFAT5‐1 (5′‐CGGACAACAAAGGCAACTCAA‐3′ sequence) or sh‐NFAT5‐2 (5′‐GCAGAGTAACTGGACGAAATA‐3′ sequence) into cells. After 48 hours of transfection, the transfection efficiency of sh‐lnc‐ISG20 and sh‐NFAT5 was determined by RT‐qPCR. AAV vectors expressing oe‐lnc‐ISG20, oe‐NFAT5, oe‐NC, shRNAs, sh‐NC and plasmids miRNA mimic, miRNA inhibitor and corresponding NCs were purchased from Shanghai GenePharma Co., Ltd (Shanghai, China).^31^, ^32^

### 2.11 RT‐qPCR

Total RNA content was extracted using Trizol kits (15 596 026, Invitrogen, Car, USA), and RNA was reverse transcribed into cDNA using reverse transcription kit (RR047A, Takara, Japan) with the volume of 20 μL. Reaction conditions were as follows: 37°C, 15 minutes; 85°C, 5 s. The SYBR Premix EX Taq kit (RR420A, Takara) was used for loading. The samples were subjected to qPCR reaction in a real‐time fluorescence quantitative PCR instrument (ABI7500, ABI, Foster City, CA, USA). Reaction system was as follows: SYBR Mix 9 μL, positive primer 0.5 μL, negative primer 0.5 μL, cDNA 2 μL and RNase Free dH_2_O 8 μL. Reaction conditions are as follows: 95°C for 10 minute, 95°C for 15 s, 60°C for 1 minute, 40 cycles in succession. Three replicate wells were set for each sample. The primers for lnc‐ISG20, miR‐486‐5p, GAPDH and U6 were synthesized by Shanghai Sangon Biotech Co., Ltd (primer sequences are shown in Table 1). The Ct value of each well was recorded, and the relative expression of the product was calculated by using the formula 2^‐ΔΔCt^ method, GAPDH and U6 were used as internal parameters. ΔΔCt= (average Ct value of target gene in experimental group‐average Ct value of housekeeping gene in experimental group)—(average Ct value of target gene in control group‐average Ct value of housekeeping gene in control group). Each experiment was repeated three times.^24^, ^33^, ^34^, ^35^

**TABLE 1:** Primer sequences

Gene	Sequence
lnc‐ISG20	F: 5′‐TTGCATCCCAGCCCTATTCCTG‐3′
	R: 5′‐GCACCCTTGACATTTCCTGTACC‐3′
NFAT5	F: 5′‐ACCATGTTCCAGTCACAGCAC‐3′
	R: 5′‐GTAAAACGACGGCCAGTAAAGTAGCCTGTGGTTGAGGC‐3′
GAPDH	F: 5′‐AGGTCGGTGTGAACGGATTTG‐3′
	R: 5′‐TGTAGACCATGTAGTTGAGGTCA‐3′
miR‐486‐5p	F: 5′‐GCATCCTGTACTGAGCTGCCC‐3′
	R: 5′‐GTATCCTGTACTGAGCTGCCC‐3′
U6	F: 5′‐CTCGCTTCGGCAGCACATATACT‐3′
	R: 5′‐ACGCTTCACGAATTTGCGTGTC‐3′

### 2.12 Western blot assay

Cells in each group were collected following digestion with trypsin, and then lysed with enhanced radio‐immunoprecipitation assay lysis buffer (Wuhan Boster Biological Technology Co., Ltd,. Wuhan, China) containing protease inhibitors. The protein concentration was detected by using bicinchoninic acid protein quantification kit (Boster). Proteins were separated by 10% sodium dodecyl sulphate‐polyacrylamide gel electrophoresis and electrotransferred to polyvinylidene fluoride membrane. Afterwards, 5% bovine serum albumin was added for incubation at room temperature for 2 hours to block non‐specific binding. Diluted rabbit anti‐collagen IV (1:1000, ab6586), rabbit anti‐fibronectin (1:1000, ab2413), rabbit anti‐TGF‐β1 (1:1000, ab92486), rabbit anti‐NFAT5 (1:1000, ab3446), rabbit anti‐p‐AKT (1:1000, ab8933), rabbit anti‐AKT (1:1000, ab179463), rabbit anti‐p‐GSK3β (1:1000, ab68476), rabbit anti‐GSK3β (1:1000, ab32391), Rabbit anti‐GAPDH (1:1000, EPR16891), all procured from Abcam, were incubated with the membrane overnight at 4°C. After washing 3 times with TBST for 5 minutes each time, HRP‐labelled goat anti‐rabbit secondary antibody (1:2000, ab205719, Abcam) was added and incubated for 1 hours at room temperature. The membrane was reacted with enhanced chemiluminescence solution (ECL808‐25, Biomiga, USA) for 1 minute at room temperature after 3 another 3 TBST rinses. Subsequently, the membranes were observed under a gel imager. GAPDH was used as an internal reference, and the ratio of the grey value of the target band to the internal reference band was used as the relative expression level of protein. Each experiment was repeated 3 times.^34^

### 2.13 RNA immunoprecipitation (RIP)

RIP assay was performed using EZ‐Magna RIP kits (17‐701, Millipore, Billerica, MA, USA) according to the manufacturer's instructions. Cells were lysed with RIP lysis buffer containing a protease and phosphatase inhibitor cocktail (Sigma‐Aldrich). Magnetic beads were pre‐incubated (Invitrogen) with AGO2 antibody (ab32381, 1:30, Abcam) or anti‐rabbit IgG (ab6721, 1:30, Abcam) for 30 minutes, immunoprecipitated with the lysate, and spined at 4°C overnight. RNA was purified from the RNA‐protein complex bound to the magnetic beads and then analysed by RT‐qPCR.^36^

### 2.14 Dual‐luciferase reporter gene experiment

Result of online website and sequence alignment indicated that lnc‐ISG20 was most likely to bind to miR‐486‐5p at two sites. The upstream and downstream sequences about 500 bp containing miR‐486‐5p potential binding site were synthesized. The wild‐type and mutant form of binding site were cloned into the psiCHECK‐2 vector R‐Luciferase (hRLuc) 3'UTR region, and then the mimics/NC of the tested miR were co‐transfected into 293T cells. The transfection method was the same as stated above. The cells were collected and lysed after 48 hours transfection. A dual‐luciferase reporter system (Jikai Gene, China) was used to detect luciferase activity. Firefly luciferase (hLuc^+^) was used as a calibration internal reference to calibrate the transfection efficiency between different samples. The ratio of RLU divided by the measured value of firefly luciferase RLU is used to evaluate the activation degree of the target reporter gene. Each experiment was repeated 3 times.^37^

### 2.15 FISH

FISH technology was applied to identify the subcellular localization of lnc‐ISG20 and miR‐486‐5p. According to the instructions of RiboTM lncRNA FISH Probe Mix (Red) (C10920, Ruibo Bio, China), a cell slide was plated in a 24‐well culture plate and inoculated with cells at 6 × 10^4^/well. Upon 60%‐70% confluence, cells were fixed by 1 mL 4% paraformaldehyde at room temperature for 10 minutes, washed and reacted with 1 mL of pre‐chilled permeabilization solution (PBS containing 0.5% Triton X‐100) each well at 4°C for 5 minutes. 200 μL of pre‐hybridization solution was added to each well and blocked at 37°C for 30 minutes. The cells were incubated with 250 μL of hybridization solution at 37°C overnight in the dark. The next steps should also be performed in dark conditions. The cells were rinsed at 42°C with Buffer I (4 × SSC, 0.1% Tween‐20), Buffer II (2 × SSC), Buffer III (1 × SSC) and 1 × PBS in successively with 5 minutes each time. The cells were stained with DAPI (1:800) for 10 minutes, washed and sealed with nail polish. A total of 5 different fields of view were selected for observation and photographing under a fluorescence microscope (Olympus, Japan).^38^

### 2.16 Statistical analysis

Statistical analyses were performed using SPSS 21.0 (IBM, USA) statistical software. Measurement data were presented as mean ± standard deviation. Comparisons between two groups were analysed by the independent sample t test, and comparisons between multiple groups were analysed by the one‐way or repeated measurement analysis of variance followed by Tukey's post hoc test. Pearson correlation coefficient was used to observe the correlation of indicators. A value of *P* < .05 was considered statistically significant.

## 3 RESULTS

### 3.1 LncRNA lnc‐ISG20 is highly expressed in DN patients, DN mouse kidney tissues and cell models

**FIGURE 1:**
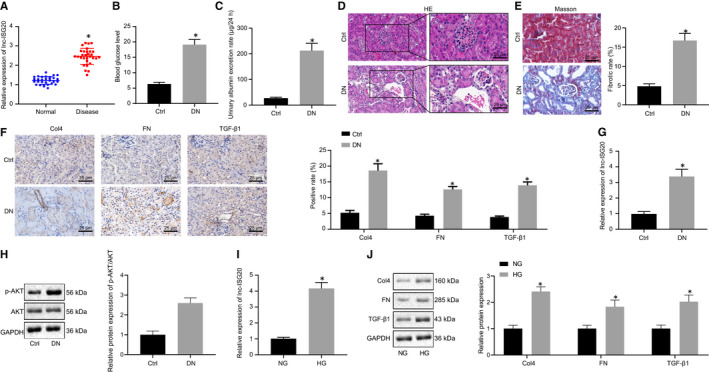
Lnc‐ISG20 is highly expressed in DN patients, DN mouse kidney tissues and cell models. A, RT‐qPCR determination of the relative expression of lnc‐ISG20 in the normal kidney tissues of the control group (n = 30) and kidney tissues of DN patients (n = 30). B, Blood glucose levels in the control and DN mice. C, Urinary albumin excretion rate in the control and DN mice. D, HE staining showing pathological conditions of the kidney tissues in the control and DN mice. E, Masson staining displaying collagen fibres of kidney tissues in the control and DN mice. F, Immunohistochemistry for detection of protein expression of collagen IV, fibronectin and TGF‐β1 in the kidney tissues of the control and DN mice. G, RT‐qPCR determination of the relative expression of lnc‐ISG20 in kidney tissues of control mice (n = 10) and DN mice (n = 10). H, The expression of p‐AKT and total AKT proteins in kidney tissues of control mice (n = 10) and DN mice (n = 10) measured by Western blot assay. I, RT‐qPCR determination of the expression of lnc‐ISG20 in MCs under NG or HG condition. J, The protein expression of collagen IV, fibronectin and TGF‐β1 in MCs under NG or HG condition measured by Western blot assay. *P < .05 when compared with the normal tissues, the control mice or NG‐treated MCs (The results are measurement data expressed by mean ± standard deviation. An independent sample t test is used for the analysis between two groups, and the experiment is repeated 3 times)

Prior research indicated that lncRNA lnc‐ISG20 was up‐regulated in the glomeruli of DN patients.^24^ In order to confirm the dysregulation of lnc‐ISG20 in DN, RT‐qPCR was conducted to detect the expression patterns of lnc‐ISG20 in clinical kidney tissues of DN patients. The results showed that lnc‐ISG20 was markedly elevated in the kidney tissues of DN patients when compared with the controls (Figure 1A).

In order to further investigate the dysregulation of lnc‐ISG20 in DN, we established DN mouse models. Compared with the control group, it was found that the blood glucose and excretion rate of urinary albumin in the DN group were increased significantly (Figure 1B, 1C). HE staining results showed intact base of kidneys in normal mice without glomerular, interstitial hyperplasia or fibrous tissue hyperplasia. Meanwhile, proliferation of mesangial cells, increased mesangial extracellular matrix, thickening of the basement membrane, presence of interstitial inflammatory cells, and hyperplasia and infiltration in fibrous tissues were observed in the DN group (Figure 1D). In addition, Masson staining illustrated a large amount of collagen deposition (blue) in the kidney tissues of the DN group (Figure 1E). Results of immunohistochemistry showed that the expression levels of collagen IV, fibronectin and TGF‐β1 in the DN group were all significantly increased relative to the control group (Figure 1F). These results demonstrated that the occurrence of kidney fibrosis in the kidneys of DN mice, indicating successful construction of DN mouse models.

Expression patterns of lnc‐ISG20 in the kidneys of DN mice were detected using RT‐qPCR, which revealed that the expression levels of lnc‐ISG20 in the DN group were significantly increased when compared with the control group (Figure 1G). Meanwhile, the Western blot assay results demonstrated that p‐AKT/AKT ratio was elevated in the kidney tissues of DN mice as compared to that of control mice (Figure 1H). In addition, NG and HG were applied to treat MCs to simulate the growth environment of MCs under normal conditions and DN conditions. Results of RT‐qPCR showed that the expression levels of lnc‐ISG20 were also significantly increased in the HG‐treated MCs (Figure 1I). The expression of collagen IV, fibronectin and TGF‐β1 was increased in the HG‐treated MCs relative to the NG‐treated MCs (Figure 1J).

### 3.2 Lnc‐ISG20 promotes the fibrosis of MCs

**FIGURE 2:**
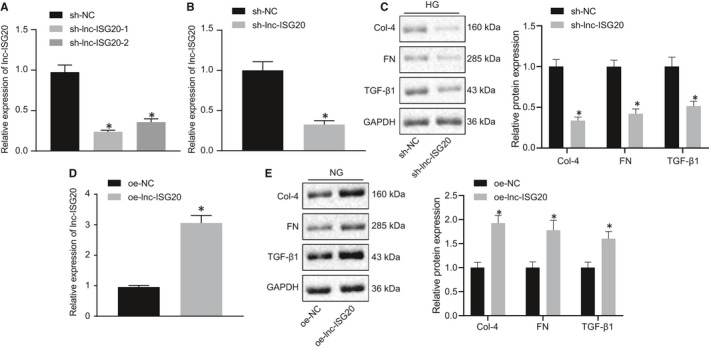
Lnc‐ISG20 promotes fibrosis of MCs. A, The efficiency of different sh‐lnc‐ISG20 sequences in MCs determined by RT‐qPCR. B, RT‐qPCR determination of lnc‐ISG20 expression in MCs after treatment with sh‐lnc‐ISG20. C, Western blot assay for the protein expression of collagen IV, fibronectin and TGF‐β1 in MCs after lnc‐ISG20 silencing. D, The efficiency of oe‐lnc‐ISG20 in MCs cells determined by RT‐qPCR. E, Western blot assay for the protein expression of collagen IV, fibronectin and TGF‐β1 in MCs after lnc‐ISG20 overexpression. *P < .05 when compared with the sh‐NC or oe‐NC group (the results are measurement data expressed by mean ± standard deviation is used for expression. An independent sample t test is used for the analysis between two groups. One‐way analysis of variance was used to analyse the data among multiple groups, followed by Tukey's post hoc test, and the experiment was repeated 3 times)

In order to explore the effect of lnc‐ISG20 on renal interstitial fibrosis, two sh‐lnc‐ISG20 sequences were designed and RT‐qPCR was performed to detect their efficiency in MCs. Compared with the sh‐NC group, the expression levels of lnc‐ISG20 in the sh‐lnc‐ISG20 group were found to be significantly reduced, and sh‐lnc‐ISG20‐1 exhibited the most obvious silencing effect, so sh‐lnc‐ISG20‐1 was selected for subsequent experimentation (Figure 2A). MCs were transfected with sh‐NC, or sh‐lnc‐ISG20 under HG treatment conditions. Western blot results revealed that compared with the sh‐NC group, the expression levels of collagen IV, fibronectin and TGF‐β1 in the sh‐lnc‐ISG20 group were all significantly reduced (Figure 2B, 2C), suggesting that inhibition of lnc‐ISG20 blocked HG‐induced fibrosis in MCs. After transfecting MCs under NG conditions with oe‐lnc‐ISG20, lnc‐ISG2 expression was significantly increased (Figure 2D). Consequently, the expression levels of collagen IV, fibronectin and TGF‐β1 were all significantly increased when lnc‐ISG2 was overexpressed in the NG‐treated MCs (Figure 2E). These data further supported the potential role of lnc‐ISG20 in promoting fibrosis of MCs.

### 3.3 Silencing of lnc‐ISG20 in kidney tissues inhibits renal fibrosis in DN mice

**FIGURE 3:**
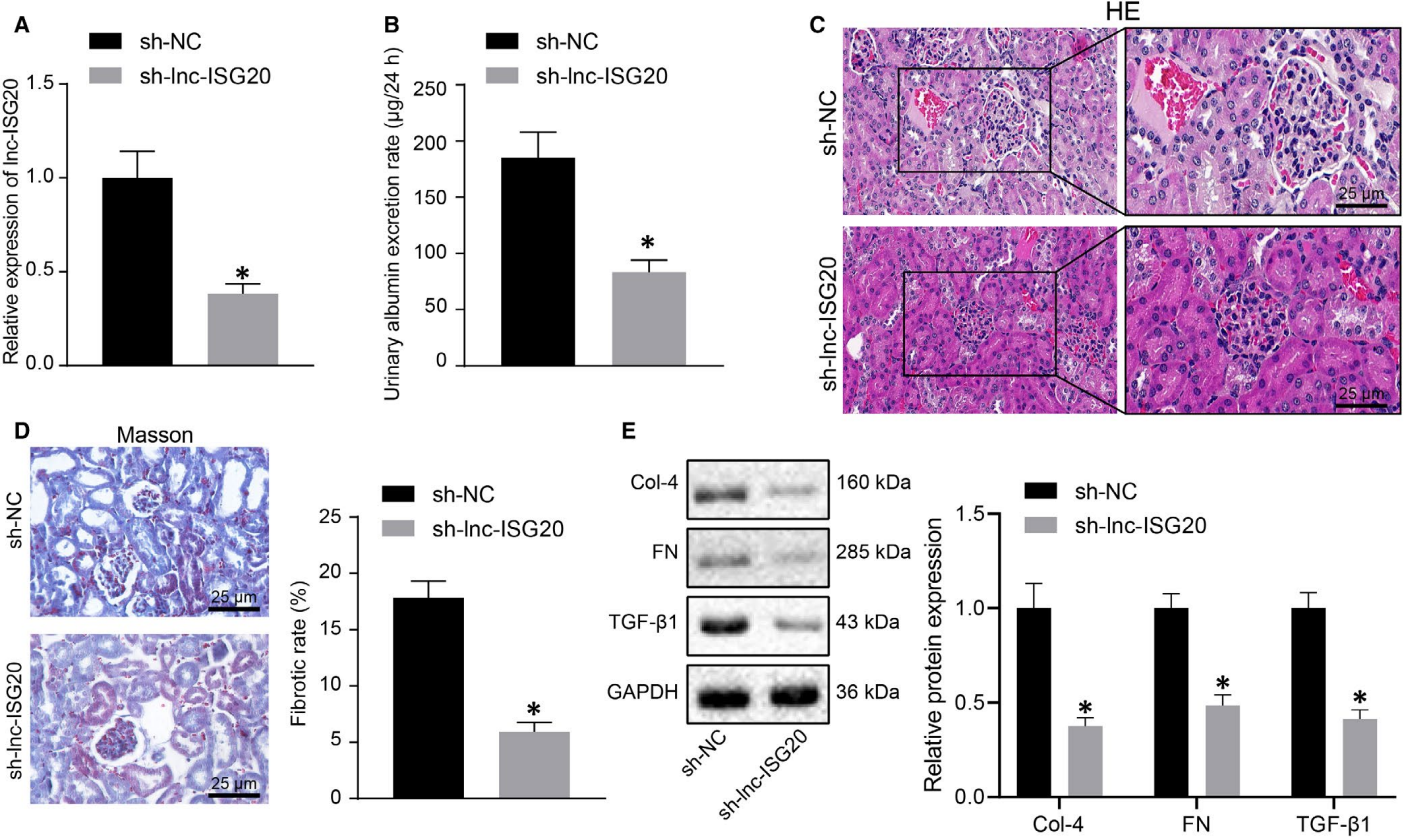
Lnc‐ISG20 knockdown in kidney tissue of DN mice can inhibit renal fibrosis. A, RT‐qPCR determination of the knockdown efficiency of AAV vector expressing sh‐lnc‐ISG20 in kidney tissues of DN mice. B, The urine albumin excretion rate in DN mice after lnc‐ISG20 knockdown. C, HE staining showing the pathological conditions of kidney tissues in DN mice after lnc‐ISG20 knockdown. D, Masson staining displaying collagen fibres in the kidney tissue of DN mice after lnc‐ISG20 knockdown. E, Western blot assay for the expression of collagen IV, fibronectin and TGF‐β1 proteins in kidney tissues of DN mice after lnc‐ISG20 knockdown. *P < .05 when compared with sh‐NC group (The results are measurement data expressed by mean ± standard deviation. The two groups were analysed by independent sample t test, and the experiment was repeated 3 times)

To further elucidate the effect of lnc‐ISG20 on renal fibrosis in DN mice, the AAV2 vectors expressing sh‐NC and sh‐lnc‐ISG20 were delivered into the kidneys of DN mice *via* orthotopic multi‐point injection to silence lnc‐ISG20. Compared with the sh‐NC group, the expression of lnc‐ISG20 in the sh‐lnc‐ISG20 group was significantly reduced (Figure 3A). As shown in Figure 3B, lnc‐ISG20 knockdown reduced the urinary albumin excretion rate in DN mice. HE and Masson staining results exhibited that the proliferation of glomerular mesangial cells, hyperplasia of glomeruli and interstitium and fibrosis were alleviated by lnc‐ISG20 knockdown (Figure 3C) and collagen deposition (blue) was significantly reduced after lnc‐ISG20 knockdown (Figure 3D), indicating that lnc‐ISG20 knockdown delayed DN‐related renal fibrosis. As expected at molecular levels, the expression levels of collagen IV, fibronectin and TGF‐β1 in the sh‐lnc‐ISG20 group were all found to be significantly lower than those in the sh‐NC group (Figure 3E). These findings suggested the inhibitory role of lnc‐ISG20 silencing in renal fibrosis in DN mice.

### 3.4 Lnc‐ISG20 binds to miR‐486‐5p

**FIGURE 4:**
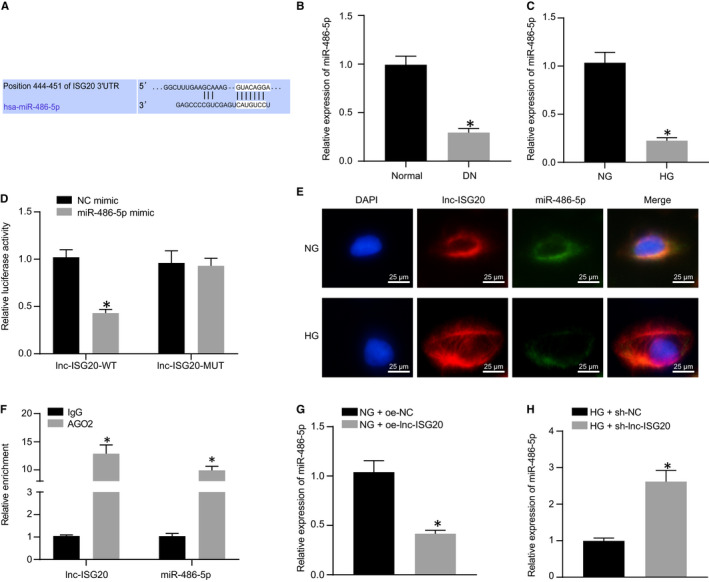
miR‐486‐5p is a downstream miRNA of lnc‐ISG20. A, Targetscan showing the binding of lnc‐ISG2 to miR‐486‐5p. B, The relative expression of miR‐486‐5p in kidney tissues of normal mice (n = 10) and DN mice (n = 10) determined by RT‐qPCR; C, The expression of miR‐486‐5p in MCs under NG and HG treatment conditions determined by RT‐qPCR. D, Verification of the binding of lnc‐ISG20 to miR‐486‐5p by dual‐luciferase reporter assay. E, The localization of lnc‐ISG20 and miR‐486‐5p in MCs identified by FISH. F, Verification of the binding of lnc‐ISG20 and miR‐486‐5p by RIP experiment. G, Under HG treatment condition, the expression of miR‐486‐5p in MCs after lnc‐ISG20 silencing determined by RT‐qPCR. H, Under NG treatment condition, the expression of miR‐486‐5p in MCs after lnc‐ISG20 overexpression determined by RT‐qPCR. *P < .05 compared with Normal mice, NG‐treated MCs, 293T cells treated with NC mimic, MCs treated with IgG antibody, MCs treated with HG + sh‐NC or MCs treated with NG + oe‐NC (The data results are measurement data showed by mean ± standard deviation. Two groups were compared by independent sample t test. The experiment was repeated 3 times)

LncRNAs possess the ability to interact with miRNA as a competitive endogenous RNA. For miRNAs bound by lnc‐ISG2, miR‐486‐5p was found to be the most potential candidate through bioinformatics analysis (Figure 4A). To further clarify the possible correlation between lnc‐ISG20 and miR‐486‐5p, the expression of miR‐486‐5p in kidney tissue of DN mice was determined by RT‐qPCR. The expression of miR‐486‐5p was significantly reduced in kidney tissue of DN mice (Figure 4B). At the cellular level, compared with the NG‐treated MCs, the expression of miR‐486‐5p in MCs was significantly reduced under HG treatment conditions, which was negatively correlated with the expression of lnc‐ISG20 (Figure 4C). In order to verify the interrelationship between lnc‐ISG20 and miR‐486‐5p, we performed dual‐luciferase reporter analysis on HEK293T cells. The wide‐type or mutant sequence was sub‐cloned into the luciferase reporter vector psi‐CHECK2. Results showed that miR‐486‐5p mimic can significantly reduce the luciferase activity of lnc‐ISG20 wide‐type plasmid, but not affect the luciferase activity of the lnc‐ISG20 mutant plasmid, suggesting a possible direct interaction between lnc‐ISG20 and miR‐486‐5p (Figure 4D). Furthermore, FISH experiments in MCs revealed that lnc‐ISG20 and miR‐486‐5p were co‐expressed in the cytoplasm, while the expression of lnc‐ISG20 increased and that of miR‐486‐5p reduced under HG condition relative to that under NC condition (Figure 4E). Meanwhile, RIP experimentation results showed that the expression levels of lnc‐ISG20 and miR‐486‐5p in the AGO2 group were both significantly increased when compared with the IgG group, demonstrating a direct binding relation between lnc‐ISG20 and miR‐486‐5p (Figure 4F). Next, under unified HG treatment conditions, the expression of miR‐486‐5p was significant increased after lnc‐ISG20 silencing in MCs (Figure 4G). Under NG treatment conditions, the expression of miR‐486‐5p was significantly reduced when lnc‐ISG20 was overexpressed in MCs (Figure 4H). Together, these results indicated that lnc‐ISG20 could bind to miR‐486‐5p.

### 3.5 miR‐486‐5p inhibits the expression of NFAT5 by targeting its 3'UTR

**FIGURE 5:**
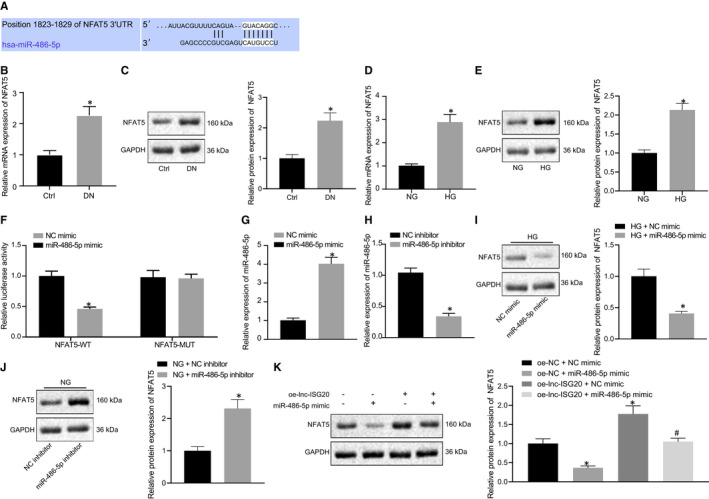
MiR‐486‐5p targets and reduces NFAT5 expression. A, Targetscan showing the binding of miR‐486‐5p to NFAT5. B, The mRNA levels of NFAT5 in kidney tissues of normal mice (n = 10) and DN mice (n = 10) determined by RT‐qPCR. C, The protein levels of NFAT5 in kidney tissues of normal mice (n = 10) and DN mice (n = 10) measured by Western blot assay. D, The mRNA levels of NFAT5 in MCs under NG and HG treatment conditions determined by RT‐qPCR. E, The protein levels of NFAT5 in MCs under NG and HG treatment conditions measured by Western blot assay. F, Verification of the binding of miR‐486‐5p to NFAT5 by dual‐luciferase reporter assay. G, The expression of miR‐486‐5p in MCs after miR‐486‐5p mimic treatment determined by RT‐qPCR. H, The expression of miR‐486‐5p in MCs after miR‐486‐5p inhibitor treatment determined by RT‐qPCR. I, Under HG condition, the mRNA levels of NFAT5 in MCs after miR‐486‐5p overexpression. J, Under NG condition, the mRNA levels of NFAT5 in MCs after inhibition of miR‐486‐5p. K, The protein levels of NFAT5 in MCs after treatment with oe‐lnc‐ISG20/oe‐NC + miR‐486‐5p mimic/NC mimic. *P < .05 compared with Ctrl mice, 293T cells treated with NG, MCs treated with NC mimic, MCs treated with NC inhibitor or MCs treated with oe‐NC + NC mimic. #*P* < .05 compared with MCs treated with oe‐lnc‐ISG20 + NC mimic (The data results are measurement data expressed by mean ± standard deviation. Independent sample t test was used for analysis between two groups, while one‐way analysis of variance was used for multiple groups followed by Tukey's post hoc test. The experiment was repeated 3 times)

It is well‐known that miRNA can regulate gene expressions by binding to the 3'‐UTR of mRNA. In order to reveal the possible regulatory loop among lncRNA, miRNA and mRNA in DN, we analysed the potential target genes of miR‐486‐5p and found a miR‐486‐5p binding site in the 3′‐UTR region of NFAT5 (Figure 5A). The expression of NFAT5 in normal mice and DN mice was measured by RT‐qPCR and Western blot analyses. The results showed that the expression of NFAT5 in DN mice was significantly increased as compared to normal mice (Figure 5B, 5C). In addition, compared with NG‐treated MCs, the expression of NFAT5 in MCs under HG treatment was significantly increased (Figure 5D). Meanwhile, the results of a luciferase assay showed that miR‐486‐5p mimic significantly reduced the luciferase activity of the NFAT5 wild‐type plasmid, but did not alter the luciferase activity of the NFAT5 mutant plasmid, suggesting that miR‐486‐5p may have a direct interaction with NFAT5 (Figure 5F). In addition, we elevated the expression of miR‐486‐5p using miR‐486‐5p mimic (Figure 5G), and reduced its expression using miR‐486‐5p inhibitor in MCs (Figure 5H). Furthermore, elevation of miR‐486‐5p caused a reduction in NFAT5 expression (Figure 5I), while inhibition of miR‐486‐5p markedly promoted the expression of NFAT5 (Figure 5J). To further explore whether lnc‐ISG20 exerted its biological function through the miR‐486‐5p/NFAT5 axis, we designed a rescue experiment and found that overexpression of lnc‐ISG20 could notably increase the protein expression levels of NFAT5 in MCs, which could be reversed by miR‐486‐5p mimic (Figure 5K). Therefore, these results indicated that lnc‐ISG20 up‐regulated the expression of NFAT5 through functioning as a ceRNA of miR‐486‐5p in MCs.

### 3.6 NFAT5 promotes fibrosis of MCs by inducing AKT phosphorylation

**FIGURE 6:**
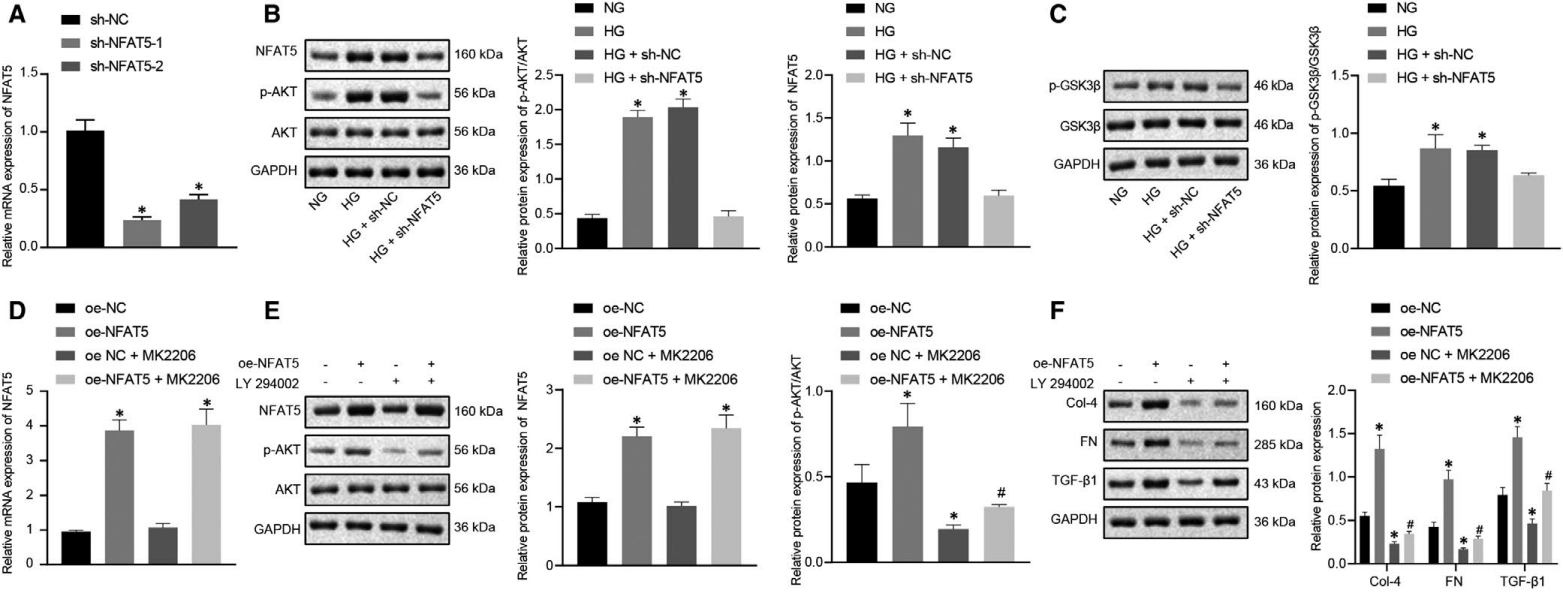
NFAT5 enhances fibrosis of MCs by activating AKT. A, The mRNA levels of NFAT5 in MCs after treatment with sh‐NFAT5 determined by RT‐qPCR. B, The protein expression of NFAT5, p‐AKT and AKT in MCs after treatment with sh‐NFAT5 under HG condition measured by Western blot assay. C, The protein expression of p‐GSK3β and GSK3β in MCs after treatment with sh‐NFAT5 under HG condition measured by Western blot assay. D, The mRNA levels of NFAT5 in MCs after treatment with oe‐NFAT5 + AKT inhibitor under HG condition determined by RT‐qPCR. E, The protein expression of NFAT5, p‐AKT and AKT in MCs after treatment with oe‐NFAT5 + AKT inhibitor under HG condition measured by Western blot assay. F, The protein expression of collagen IV, fibronectin and TGF‐β1 in MCs after treatment with oe‐NFAT5 + AKT inhibitor under HG condition measured by Western blot assay. *P < .05 compared with MCs treated with NG, sh‐NC or oe‐NC. #*P* < .05 compared with MCs treated with oe‐NFAT5 (The data results are measurement data expressed by mean ± standard deviation. One‐way analysis of variance was used for comparison among multiple groups followed by Tukey's post hoc test, and the experiment was repeated 3 times)

NFAT5 has been previously demonstrated to interact with multiple signalling pathways.^39^ Considering that the AKT pathway is activated in the DN rat model, which shares a relationship with renal fibrosis,^40^ the activation of AKT pathway could be a possible target to affect the renal fibrosis in DN. Therefore, we tested whether NFAT5 would affect the activation of AKT. We silenced NFAT5 in MCs using two sh‐NFAT5 sequences. Due to the efficiency of sh‐NFAT5‐1 was higher, sh‐NFAT5‐1 was selected for subsequent experiments (Figure 6A). Compared with NG‐treated MCs, the expression of NFAT5 and the extent of AKT phosphorylation in HG‐treated MCs were increased, both of which were reduced when NFAT5 was silenced; however, the total AKT protein expression remained unchanged (Figure 6B). Furthermore, we assessed the phosphorylation of GSK3β, which is one of phosphorylated substrates of AKT. Similarly, the phosphorylation of GSK3β was activated in MCs under HG condition, which was inhibited by sh‐NFAT5, while the total GSK3β protein expression remained unchanged (Figure 6C). These results indicated that NFAT5 enhanced the phosphorylation of AKT in MCs.

To investigate whether NFAT5 regulates AKT phosphorylation to affect fibrosis of MCs, the AKT inhibitor MK2206 (25 μM) and oe‐NFAT5 were used at the same time under the HG condition. RT‐qPCR results validated the overexpression efficiency of oe‐NFAT5 (Figure 6D). Western blot data suggested that the protein NFAT5 expression and the extent of AKT phosphorylation were appreciably increased by oe‐NFAT5, while the enhanced AKT phosphorylation was reversed by MK2206 (Figure 6E). Also, the results showed that oe‐NFAT5 could increase the expression of collagen IV, fibronectin and TGF‐β1 in HG‐treated MCs. However, MK2206 could attenuate the oe‐NFAT5‐induced increases of collagen IV, fibronectin and TGF‐β1 (Figure 6F). These data suggested that NFAT5 could promote the fibrosis of MCs by stimulating HG‐induced AKT phosphorylation.

### 3.7 Lnc‐ISG20 promotes fibrosis of MCs through miR‐486‐5p/NFAT5/p‐AKT

**FIGURE 7:**
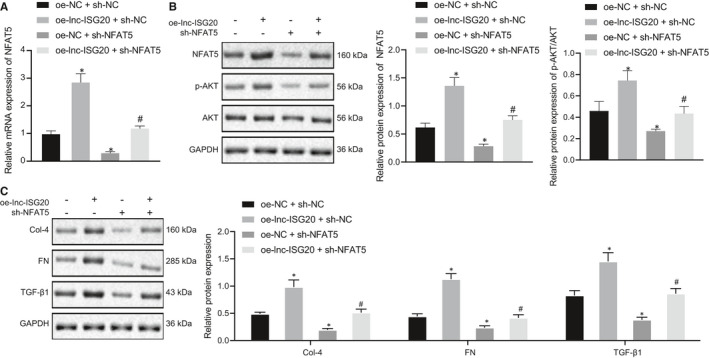
Lnc‐ISG20 promotes fibrosis of MCs through miR‐486‐5p/NFAT5/p‐AKT. A, The mRNA levels of NFAT5 in MCs after treatment with oe‐lnc‐ISG20/oe‐NC + sh‐NC/sh‐NFAT5 determined by RT‐qPCR. B, The expression of NFAT5, p‐AKT, AKT in MCs after treatment with oe‐lnc‐ISG20/oe‐NC + sh‐NC/sh‐NFAT5 determined by RT‐qPCR. C, The protein expression of collagen IV, fibronectin and TGF‐β1 in MCs under unified HG treatment conditions after treatment with oe‐lnc‐ISG20/oe‐NC + sh‐NC/sh‐NFAT5 measured by Western blot assay. *P < .05 as compared to MCs treated with oe‐NC + sh‐NC. #*P* < .05 as compared to the MCs treated with oe‐lnc‐ISG20 + sh‐NC. (The data results are measurement data expressed by mean ± standard deviation. One‐way analysis of variance was used for comparisons among multiple groups followed by Tukey's post hoc test. The experiment was repeated 3 times)

Our initial findings indicated that overexpression of lnc‐ISG20 could augment the expression of NFAT5, which could be reversed by miR‐486‐5p mimic. In order to explore whether lnc‐ISG20 exerts its biological function through the miR‐486‐5p/NFAT5/AKT axis, we designed a rescue experiment. The mRNA level of NFAT5 was significantly increased by lnc‐ISG20 overexpression but reduced by sh‐NFAT5 in the presence of lnc‐ISG20 (Figure 7A). Consequently, lnc‐ISG20 overexpression enhanced AKT phosphorylation while NFAT5 knockdown inhibited AKT phosphorylation. Furthermore, the enhanced phosphorylation of AKT induced by oe‐lnc‐ISG20 was dramatically reduced by sh‐NFAT5 (Figure 7B).

Meanwhile, the expression levels of collagen IV, fibronectin and TGF‐β1 were found to be remarkably increased by enforced expression of lnc‐ISG20, but reduced by silencing of NFAT5. The increases of collagen IV, fibronectin and TGF‐β1 caused by oe‐lnc‐ISG20 were counteracted by sh‐NFAT5 (Figure 7C). Overall, these findings indicated that lnc‐ISG20 could stimulate cell fibrosis in MCs through the miR‐486‐5p/NFAT5/p‐AKT axis.

### 3.8 Lnc‐ISG20 promotes renal fibrosis in DN mice through miR‐486‐5p/NFAT5/p‐AKT

**FIGURE 8:**
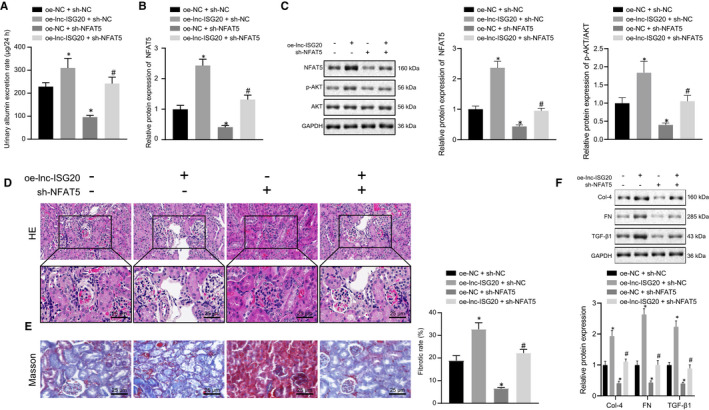
Lnc‐ISG20 promotes renal fibrosis in DN mice through miR‐486‐5p/NFAT5/p‐AKT. A, Urinary albumin excretion rate of DN mice after lnc‐ISG20 overexpression and/or NFAT5 silencing together. B, RT‐qPCR determination of the expression of NFAT5 in the kidney tissues of DN mice after lnc‐ISG20 overexpression and/or NFAT5 silencing together. C, Western blot analysis for the protein expression of NFAT5, p‐AKT and AKT in kidney tissues of DN mice after lnc‐ISG20 overexpression and/or NFAT5 silencing together. D, HE staining showing the pathological conditions of kidney tissues of DN mice after lnc‐ISG20 overexpression and/or NFAT5 silencing together. E, Masson staining displaying the collagen fibres in the kidney tissues of DN mice after lnc‐ISG20 overexpression and/or NFAT5 silencing together. F, Western blot analysis for the protein expression of collagen IV, fibronectin and TGF‐β1 in mouse kidney tissues of DN mice after lnc‐ISG20 overexpression and/or NFAT5 silencing together. *P < .05 as compared to the oe‐NC + sh‐NC group. #*P* < .05 as compared to the oe‐lnc‐ISG20 + sh‐NC group (The data results are measurement data shown by mean ± standard deviation. One‐way analysis of variance was used for comparisons among multiple groups followed by Tukey's post hoc test, and the experiment is repeated 3 times)

In order to further verify the aforementioned results, multi‐point injection in situ of AAV2 vectors was performed in vivo. The results showed increased urinary albumin excretion rate of DN mice after lnc‐ISG20 overexpression and reduced urinary albumin excretion rate of DN mice after NFAT5 knockdown. Additionally, the urinary albumin excretion rate increased by oe‐lnc‐ISG20 was markedly reduced by sh‐NFAT5 (Figure 8A). RT‐qPCR and Western blot results showed that NFAT5 was significantly increased at mRNA and protein levels upon lnc‐ISG20 overexpression, but the extent of AKT phosphorylation was reduced in response to NFAT5 knockdown (Figure 8B and 8C). However, the enhancement of AKT phosphorylation caused by lnc‐ISG20 overexpression could be reversed by NFAT5 silencing.

To further assess pathological condition of kidney tissues of DN mice after intervention, HE staining was conducted. The results demonstrated that the proliferation of mesangial cells and mesangial extracellular matrix was increased and the basement membrane became thickened after lnc‐ISG20 overexpression, accompanied with interstitial inflammatory cells and hyperplasia and infiltration in fibrous tissues of DN mice. The intact kidney base was observed without glomeruli, interstitial hyperplasia, and hyperplasia in fibrous tissues when NFAT5 was knocked down in DN mice. It was further indicated that inhibition of NFAT5 reversed the pathological conditions such as renal fibrosis caused by oe‐lnc‐ISG20 (Figure 8D). In addition, Masson staining showed that increased collagen fibre deposition caused by lnc‐ISG20 overexpression was reversed by sh‐NFAT5 (Figure 8E). The expression of collagen IV, fibronectin and TGF‐β1 in kidney tissues of DN mice was also measured. We found that the expression levels of collagen IV, fibronectin and TGF‐β1 were all increased when lnc‐ISG20 was overexpressed, but reduced when NFAT5 was silenced in DN mice. The up‐regulation of collagen IV, fibronectin and TGF‐β1 caused by lnc‐ISG20 overexpression was reversed by NFAT5 knockdown (Figure 8F). Hence, these findings indicated that lnc‐ISG20 stimulated AKT phosphorylation and promoted renal fibrosis in DN mice by inducing NFAT5.

## 4 DISCUSSION

Currently, microalbuminuria evaluation is regarded as the gold‐standard for the diagnosis of early renal involvement, however, the unsatisfactory efficacy of urine testing exists as it can be precipitated by many common clinical factors, such as acute febrile disease, exercise and transient loss of glycemic control.^41^ On the other hand, growing evidence has implicated the dysregulation of miRNAs as well as lncRNAs in the pathogenesis of DN.^11^, ^16^ One such lncRNA, lnc‐ISG20 was documented to exhibit up‐regulated expression levels in the glomerular of DN patients, while miR‐486‐5p was correlated with blood glucose or albuminuria, all of which enable non‐coding RNAs to serve as promising biomarkers for DN or renal fibrosis.^7^, ^24^ However, the underlying mechanisms of lnc‐ISG20 and miR‐486‐5p in regard to DN remain to be uncovered. In view of this, the current study performed a series of experiments, and our findings demonstrated that lnc‐ISG20 was highly expressed in DN patients, and exhibited a close relationship with renal fibrosis. Additionally, the expressions of collagen IV and fibronectin as well as TGF‐β1 induced by HG could be modulated by controlling lnc‐ISG20. Further analysis based online database and dual‐luciferase assay uncovered that lnc‐ISG20 could bind to miR‐486‐5p which could further target NFAT5. Furthermore, NFAT5 silencing could inhibit AKT phosphorylation and thus alleviate the HG‐induced fibrosis in MCs.

Firstly, our findings revealed that lnc‐ISG20 was highly expressed in DN patients, the kidney tissues in DN mice, as well as the MCs under HG conditions. The ISG20 gene possesses the ability to encode a 20‐kDa protein, which is extensively involved in processes like oncogenesis and gene transcription.^42^ In addition, ISG20 is often associated with immune responses, accompanied by increased macrophage count and neutrophil infiltration in tumours.^43^ Chai *et al* firstly identified lnc‐ISG20 as long non‐coding RNA and characterized its positive regulatory effects on the protein levels of ISG20.^44^ Moreover, a recent study has also found elevated expression levels of lnc‐ISG20 in the glomerulus of DN patients,^24^ which is consistent with the findings uncovered in our study. Moreover, the high‐expression pattern of lnc‐ISG20 in DN and its correlation with renal fibrosis indicate that lnc‐ISG20 can serve as a promising biomarker for the renal fibrosis in DN.

Additionally, the current study demonstrated that lnc‐ISG20 could promote cell fibrosis in MCs, while silencing of lnc‐ISG20 in DN mice brought about inhibitory effects on renal fibrosis. The progression of DN is commonly characterized by the loss of renal cells, and renal fibrosis represents the final pathological change in DN.^45^ So far, there is a glaring lack of treatment strategies to prevent the progression of renal fibrosis, hence, revealing the molecular mechanism could prove to be highly valuable in progressing the treatment of renal fibrosis or DN.^46^ More notably, several studies have highlighted the ability of lncRNAs to modulate cell proliferation and fibrosis in DN, including lncRNA Erbb4,^47^ lnc‐TSI,^48^ lncRNA 1700020I14Rik^28^ and so on. Herein, our findings illustrated that lnc‐ISG20 could promote fibrosis, thus enriching the palette of regulatory lncRNAs in DN. Further experimentation in our study revealed that lnc‐ISG20 knockdown could reduce the expression levels of collagen IV, fibronectin and TGF‐β1, among which TGF‐β is the primary factor that drives fibrosis in almost all forms of chronic kidney disease. In addition, as the master regulator of fibrosis, TGF‐β can induce the activation of myofibroblasts, excessive production of ECM and inhibition of ECM degeneration.^49^ Overall, these findings and evidence suggest that lnc‐ISG20 could serve the upstream molecule of TGF‐β to perform the modulatory functions, which also highlights a potential approach to manipulate the progression of fibrosis.

Furthermore, the current study revealed that lnc‐ISG20 was capable of binding to miR‐486‐5p. Meanwhile, numerous studies have documented the tumour suppressive role of miR‐486‐5p in several kinds of malignancies, such as lung squamous cell carcinoma, non‐small cell lung cancer and breast cancer.^50^, ^51^, ^52^ Besides, miR‐486‐5p was also correlated with albuminuria in DN by a previous study, wherein miR‐586‐5p could regulate oxidative stress, inflammation, as well as apoptosis.^13^ Expanding on this role, we identified that lnc‐ISG20 served as an upstream regulator for miR‐486‐5p in DN, in which lnc‐ISG20 could down‐regulate the expression of miR‐486‐5p and thus relieve its inhibitory effect, resulting in the promotion of renal fibrosis. Various authors have shed a great deal of light on the phenomenon by which lncRNAs can modulate fibrosis *via* binding to miRNAs. For instance, lncRNA1700020l14Rik could interact with miR‐34a‐4p to alleviate cell proliferation and fibrosis in DN.^28^ Whereas, lncRNA MALAT1 could regulate renal tubular epithelial pyroptosis in DN by modulating miR‐23c, which is similar to our findings in regard to targeting of miR‐486‐5p by lnc‐ISG20 in DN.^53^ Moreover, the current study further revealed that miR‐486‐5p could inhibit the expression of NFAT5. Several studies have characterized the relationship between miRNAs and NFAT in multiple physiological processes; for example, miR‐211/132 has been shown to affect calcineurin/NFAT signalling to regulate both cardiac hypertrophy and cardiomyocyte autophagy.^54^ Similarly, miR‐21b can inhibit T‐cell proliferation and activation through the NFAT signalling pathway.^55^ More importantly, a previous study demonstrated that the transcription factor, NFAT, can play essential roles in the early stages of DN, wherein the inhibition of calcineurin/NFAT conferred protective effects against DN.^20^ Here, we identified that miR‐486‐5p served as an upstream modulator for NFAT5 and could down‐regulate its expression. Together, our findings not only identified miR‐485‐5p as a downstream target for lnc‐ISG20, but also highlighted NFAT5 as the downstream target for miR‐485‐5p, thus consummating the signalling pathways implicated in DN.

Expanding on our discoveries, we further uncovered that overexpression of NFAT5 could trigger cell fibrosis in MCs by activating the AKT protein. AKT, also known as protein kinase B or PKB, can be activated by multiple factors such as growth factors and the oncogenic mutations of upstream regulatory proteins.^56^ Moreover, AKT is capable of regulating diverse biological processes, including cell proliferation, survival and metabolism and so forth, whereas the dysregulation of AKT may lead to cancer, cardiovascular and neurological diseases, as well as diabetes.^57^ Existent reports suggest that AKT can be modulated by several upstream regulators, like tamoxifen, Aloe‐Emodin, miRNAs, and thus regulate fibrosis in kidney, liver and heart etc.^58^, ^59^, ^60^ Additionally, AKT phosphorylation has also been previously demonstrated to be implicated in renal dysfunction, such as renal tubular cell apoptosis, renal fibrosis.^23^, ^61^, ^62^ In our study, NFAT5 was found to enhance renal fibrosis *via* inducing AKT phosphorylation, which deepens our understanding of the molecular mechanisms underlying DN. Based on all above findings, our study indicates towards the existence of a regulatory axis in the pathophysiology of DN, wherein lnc‐ISG20 promotes renal fibrosis *via* the miR‐486‐5p/NFAT5/p‐AKT pathway. The newly proposed signalling axis in the present study contains many important regulatory factors, including lncRNAs, miRNAs, transcription factors as well as protein kinases, which enriches our understanding on both non‐coding RNAs and the pathogenesis of the disease.

## 5 CONCLUSIONS

**FIGURE 9:**
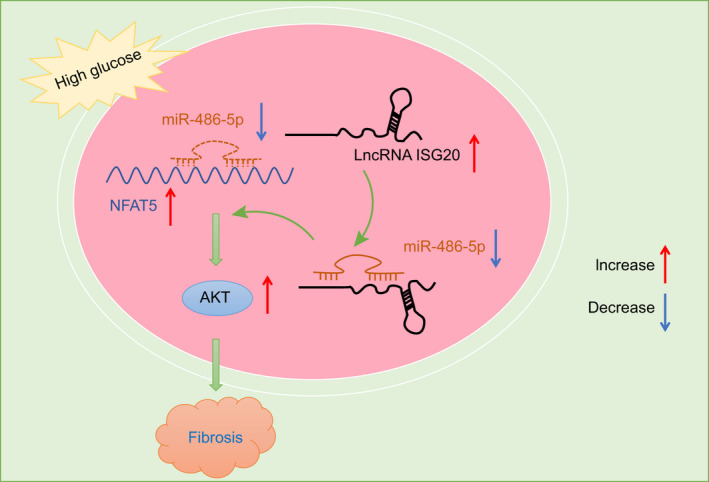
Lnc‐ISG20 promotes renal fibrosis in DN by activating AKT through miR‐486‐5p/NFAT5

From the present study, the results suggested that lnc‐ISG20 was aberrantly up‐regulated in DN. Hence, lnc‐ISG20 may be able to serve as a promising biomarker for the diagnosis of DN. Altogether, findings from the current study identified the unique miR‐486‐5p/NFAT5/p‐AKT signalling pathway in the progression of DN. Accordingly, we found that lnc‐ISG20 promoted renal fibrosis in DN through impairing miR‐486‐5p‐dependent inhibition of NFAT5 and activating AKT (Figure 9). These hallmarks also provide potential targets for the development of new drugs or therapies. In the future, it remains important to investigate the relationship between the proposed pathway and the other related pathways, so as to provide clearer route for drug or therapy development. Also, whether lnc‐ISG20 acts in cell types other than MCs such as renal tubular epithelial cells in renal fibrosis is necessary to be investigated in the future. However, it is worth noting that the broad involvement of the vital molecules such as TGF‐β, NFAT5 or AKT in many other physiological processes especially multiple mechanisms controlled by NFAT5 should be paid more attention in the follow‐up researches.

## CONFLICTS OF INTEREST

The authors declare no conflict of interest.

## AUTHOR CONTRIBUTION

Yu‐Rui Duan: Conceptualization (lead); Data curation (equal); Formal analysis (equal); Investigation (lead); Methodology (lead); Writing‐original draft (lead); Writing‐review & editing (equal). Bao‐Ping Chen: Conceptualization (supporting); Data curation (equal); Formal analysis (equal); Investigation (supporting); Methodology (supporting); Writing‐original draft (supporting); Writing‐review & editing (equal). Fang Chen: Data curation (equal); Formal analysis (equal); Project administration (lead); Resources (equal); Software (equal); Writing‐review & editing (equal). Su‐Xia Yang: Data curation (equal); Formal analysis (equal); Project administration (supporting); Software (equal); Validation (equal); Visualization (equal); Writing‐review & editing (equal). Chao‐Yang Zhu: Data curation (equal); Formal analysis (equal); Software (equal); Validation (equal); Visualization (equal); Writing‐review & editing (equal). Ya‐Li Ma: Data curation (equal); Formal analysis (equal); Software (equal); Validation (equal); Visualization (equal); Writing‐review & editing (equal). Yang Li: Data curation (equal); Formal analysis (equal); Resources (equal); Software (equal); Supervision (equal); Validation (equal); Visualization (equal); Writing‐review & editing (equal). Jun Shi: Data curation (equal); Formal analysis (equal); Funding acquisition (lead); Resources (equal); Software (equal); Validation (equal); Writing‐review & editing (equal).

## AUTHORS CONTRIBUTION

YRD, BPC, FC, SXY, CYZ, YLM, YL and JS: Study design. YRD and BPC: Data collation; data analyses; and production of the initial draft of the manuscript. FC, SXY, CYZ, YLM, YL and JS: Contribution of manuscript drafting. All authors: Read and approval of the final submitted manuscript.

## Data Availability

The datasets generated and/or analysed during the current study are available from the corresponding author on reasonable request.
